# Adverse pregnancy outcomes across SLE subgroups: significance of cardiovascular events

**DOI:** 10.1136/lupus-2025-001507

**Published:** 2025-04-29

**Authors:** Rashmi Dhital, Rebecca J Baer, Kenneth Kalunian, Christina Chambers

**Affiliations:** 1 Department of Medicine, Division of Rheumatology and Immunology, Vanderbilt University Medical Center, Nashville, Tennessee, USA; 2 Department of Pediatrics, Division of Environmental Science and Health, University of California San Diego, La Jolla, California, USA; 3 The California Preterm Birth Initiative, University of California San Francisco, San Francisco, California, USA; 4 Department of Medicine, Division of Rheumatology, University of California San Diego, La Jolla, California, USA; 5 Herbert Wertheim School of Public Health and Human Longevity Science, University of California San Diego, La Jolla, California, USA

**Keywords:** Antiphospholipid Syndrome, Cardiovascular Diseases, Epidemiology, Lupus Erythematosus, Systemic, Lupus Nephritis

## Abstract

**Objective:**

SLE is associated with increased risks of maternal cardiovascular events (CVEs) as well as adverse pregnancy outcomes. The influence of maternal CVEs on pregnancy complications in lupus is not clearly known. Our primary aim was to assess the risks of adverse pregnancy outcomes in individuals with SLE, specifically examining the influence of CVEs.

**Methods:**

Using a California population-based birth cohort from 2005 to 2020, pregnant individuals with SLE were identified via International Classification of Diseases codes on maternal discharge records and further subdivided based on whether they had lupus nephritis (LN) or antiphospholipid syndrome (APS). We analysed adjusted relative risks (aRRs) of adverse pregnancy outcomes in SLE subgroups, comparing those with and without CVEs, to the reference group of pregnant individuals without autoimmune rheumatic diseases or APS and CVEs. CVEs were broadly defined to encompass thromboembolic and cardiovascular conditions associated with SLE.

**Results:**

CVEs complicated 17 130/7004 334 (0.2%) of pregnancies in individuals without autoimmune rheumatic diseases or APS, and 176/8422 (2.1%) with SLE, including 52/903 (5.8%) with LN and 40/513 (7.8%) with APS. Compared with the reference group, the aRRs for maternal complications were higher in SLE subgroups: non-cardiac severe maternal morbidity (3.2-fold to 31.5-fold), intensive care admission (2.0-fold to 12.2-fold), 1 year re-admission (2.4-fold to 6.0-fold) and death (7.0-fold to 7.9-fold). Similarly, adverse infant outcomes were higher: preterm birth (2.3-fold to 6.8-fold), small-for-gestational-age infant (1.8-fold to 3.4-fold), neonatal intensive care admission (2.1-fold to 7.9-fold) and infant death (1.6-fold to 3.7-fold), with highest risk estimates for SLE with LN or APS, particularly when complicated by CVEs.

**Conclusions:**

LN and APS in SLE contributed to incremental risks for adverse outcomes, with the combination of LN or APS with CVEs yielding the highest point estimates. This underscores the importance of disease severity but also the impact of CVEs, helping to individualise the risks of pregnancy complications for various SLE subpopulations.

**WHAT IS ALREADY KNOWN ON THIS TOPIC**

**WHAT THIS STUDY ADDS**

- LN and APS in SLE were associated with higher risks for adverse outcomes beyond SLE without those manifestations, and maternal CVEs contributed to incremental risks of the adverse outcomes within each SLE subgroup.

**HOW THIS STUDY MIGHT AFFECT RESEARCH, PRACTICE OR POLICY**

- Our findings help to individualise the risks of pregnancy complications for various SLE subpopulations, underscoring the importance of SLE disease severity but also of CVEs on short-term as well as long-term maternal and infant outcomes.
- Future prospective studies should aim to precisely time the CVEs in relation to the stage of pregnancy and the outcomes of interest and also assess the impact of SLE medications on cardiovascular risks and pregnancy complications.

## Introduction

 SLE presents substantial risks for adverse pregnancy outcomes, including a twofold to fourfold higher risk of pre-eclampsia, preterm birth (PTB), small-for-gestational-age (SGA) infants, and neonatal intensive care unit (NICU) utilisation.^1^,^3^ These adverse outcomes correlate with disease activity and severity, presence of antiphospholipid antibodies and pre-existing comorbidities such as chronic hypertension.^4 5^

SLE is also associated with increased risk of cardiovascular events (CVEs), especially when accompanied by lupus nephritis (LN) or positive antiphospholipid antibodies.^6 7^ Notably, women aged 35–44 years with SLE may have over 50 times higher risk of myocardial infarction than age-matched controls.^8^ Studies have also shown significantly higher risks of CVEs during pregnancy and early postpartum in individuals with SLE compared with the general pregnant population.^9 10^ In our analysis of a large population-based California birth cohort, pregnant women with an autoimmune rheumatic disease (ARD) had fourfold higher risk of CVEs during pregnancy and up to 6 weeks postpartum compared with those without an ARD or antiphospholipid syndrome (APS). SLE was associated with a sixfold higher risk, which increased to 18-fold and 12-fold if SLE was accompanied by APS or LN, respectively. The majority of CVEs in SLE were venous thromboembolism (VTE, 47%), followed by heart failure/peripartum cardiomyopathy (25%) and cardiac dysrhythmias (19%).^10^

Prior studies indicate that adverse pregnancy outcomes are associated with increased risk for future cardiovascular diseases and disease-related mortality for the mother. Shared predisposing factors may contribute to both pregnancy complications and cardiovascular diseases, and pregnancy complications may be markers of underlying subclinical and clinical vascular and metabolic risk factors.^11 12^ Similar findings have been observed in SLE, where hypertensive disorders of pregnancy doubled the risk of future CVEs, defined in the study as a composite of myocardial infarction, stroke, transient ischaemic attack, unstable angina and heart failure.^13^ However, data on how maternal CVEs may influence the risk of pregnancy complications are scarce. Using the same California birth cohort, we previously found that 53.2% of pregnant individuals with an ARD who experienced CVEs during pregnancy experienced adverse outcomes (composite of PTB and SGA infants), compared with 26.6% without such events. Maternal CVEs in ARD increased the risk of PTB by 1.6-fold and SGA infant by 1.5-fold.^14^ This relationship may be particularly relevant in SLE, which is associated with significantly higher risks of both CVEs and adverse pregnancy outcomes. The primary aim of this study was to assess the risks of adverse pregnancy outcomes in individuals with SLE compared with the general population of pregnant women without an ARD or APS, specifically examining the influence of CVEs.

## Methods

### Data source

This study was a retrospective cohort study of singleton liveborn infants in California between 2005 to 2020. Infant birth and death certificates, maintained by California Vital Statistics, were linked by the University of California San Diego Study of Outcomes of Mothers and Infants (SOMI) to maternal and infant hospital discharge, emergency department and ambulatory surgery records maintained by the California Department of Healthcare Access and Information (HCAI). Diagnoses were identified using International Classification of Diseases, 9th Revision, Clinical Modification (ICD-9, 2005 through September 2015) and International Classification of Diseases, 10th Revision, Clinical Modification (ICD-10, October 2015 through 2020) codes.

### Study Population

Pregnant women with SLE were identified using ≥1 ICD-9-CM and ICD-10-CM codes (ICD-9-CM: 710.0, ICD-10-CM M32.1x, M32.8, M32.9) on discharge records during pregnancy and/or birth admission and further stratified by the presence of LN or APS. We identified LN using ≥1 ICD-code claims for SLE combined with codes for acute or chronic glomerulonephritis, renal failure, nephritis or nephrotic syndrome, or proteinuria. Similarly, SLE with APS was identified using ICD codes for both SLE and APS (online supplemental table 1). Individuals without any of the specified ICD codes associated with ARDs or APS and without CVEs comprised the reference group.

### Exposure

The primary exposure of interest was the presence of one or more maternal CVEs in SLE and SLE subgroups. Acute CVEs during pregnancy were identified using ICD codes for (1) Acute myocardial infarction; (2) Acute cerebrovascular accident (ischaemic and haemorrhagic); (3) Heart failure and peripartum cardiomyopathy; (4) Inflammatory heart disease (myocarditis and pericarditis); (5) Cardiac dysrhythmia (atrial fibrillation/flutter, ventricular arrhythmias and cardiac arrest, arrhythmia unspecified); and (6) VTE (pulmonary embolism, deep vein thrombosis) from the patient’s last menstrual period through delivery admission (online supplemental table 2).

### Outcomes

The endpoints of interest were the selected adverse maternal and child outcomes. The maternal complications were identified using ICD codes from maternal discharge records. Maternal complications studied included non-cardiac severe maternal morbidity, maternal intensive care unit (ICU) utilisation at delivery admission, maternal re-admission and maternal death within the first year of delivery. Defined by the US Centers for Disease Control and Prevention, severe maternal morbidity includes unexpected labour and delivery outcomes with serious short-term and long-term health consequences for the mother. Non-cardiac components of severe maternal morbidity include maternal complications such as acute renal failure, acute respiratory distress syndrome, disseminated intravascular coagulation, amniotic fluid embolism, blood transfusion, severe anaesthesia complications, sepsis, hysterectomy, temporary tracheostomy and ventilation (online supplemental table 3).^15^

The infant outcomes were similarly identified from infant hospitalisation and birth certificate records. Neonatal and infant outcomes studied included PTB, SGA, NICU utilisation, infant re-admissions and mortality within the first year of life. The gestational age was obtained from the best obstetric estimate on the birth certificate record. PTB was defined as delivery occurring prior to 37 weeks of gestation. PTB was further classified as spontaneous or provider-initiated PTB, and early (<32 weeks of gestation), moderate (32 weeks to <34 weeks of gestation) or late (34 weeks to <37 weeks of gestation). SGA was defined as birth weight <10th centile for gestational age and sex.^16^ Neonatal ICU admission at birth was identified from birth certificate records.

Maternal re-admissions up to 1 year were identified from the maternal hospital discharge records and infant re-admissions from the linked HCAI records. Maternal death up to 1 year of delivery was identified from the maternal hospital discharge records where discharge status reported ‘died’ or linked death certificate records from California Vital Statistics. Infant mortality up to 1 year was identified from the California Vital Statistics birth cohort files or the linked HCAI hospital discharge records where discharge status reported ‘died’.

### Covariates

Covariates were obtained from birth certificates and hospital discharge records and were categorised as follows: maternal age (<18 years, 18–34 years, 35–44 years or ≥45 years), maternal race/ethnicity (non-Hispanic white, non-Hispanic black, Hispanic, Asian and other), expected primary payer (private, public, self-pay or other), maternal education (completion of <12th, 12th or >12th grades), prepregnancy body mass index (BMI) (calculated from prepregnancy weight and height; <18.5 kg/m^2^, 18.5 kg/m^2^ to <25 kg/m^2^, 25 kg/m^2^ to <30 kg/m^2^, ≥30 kg/m^2^), and maternal comorbidities (hypertension, diabetes mellitus, hyperlipidaemia, depression and substance use disorders (online supplemental table 2).

### Statistical analyses

We studied baseline demographic and clinical characteristics of pregnant individuals in the SLE group and those without an ARD or APS (table 1). We analysed composite and individual components of the selected adverse pregnancy outcomes in pregnant individuals with SLE, further subdividing the SLE group into those with LN or APS (table 2). We used log-linear regression with a Poisson distribution to calculate adjusted relative risks (aRRs) and 95% CIs for adverse pregnancy outcomes across SLE subgroups (SLE without LN or APS, SLE with LN, SLE with APS), comparing those with and without CVEs during pregnancy, to a reference group of pregnant women without ARD or APS and without CVEs. For comparison, we also studied the occurrence of adverse outcomes in pregnant individuals without an ARD or APS but with a CVE (table 3). For cells with fewer than 11 events, cell sizes and percentages, and relative risks were not reported to comply with IRB confidentiality requirements. A Propensity Score (PS) for SLE was derived using logistic regression from select maternal sociodemographic variables (age, race/ethnicity, primary payer, maternal education, prepregnancy BMI) and clinical comorbidities (pre-existing and pregnancy-induced hypertension and diabetes mellitus, hyperlipidaemia, depression, and substance use disorders) that have been previously identified in the literature as being linked to adverse pregnancy outcomes.^17^ Adjusted relative risks (aRRs) were calculated using the single PS approach in the regression models to avoid model overfitting due to infrequent number of events in certain subgroups.

**Table 1:** Demographic and clinical characteristics of pregnant individuals without an ARD or APS, and among those with SLE (with or without CVEs during pregnancy)

SLE	No ARD/APS	Any SLE	SLE subgroups	
			SLE with CVE	SLE without CVE
	n (%)	n (%)	n (%)	n (%)
Sample	7004 334 (100.0)	8422 (100.0)	176 (100.0)	8246 (100.0)
Maternal age (years)				
<18	151 508 (2.2)	47 (0.6)	*	46 (0.6)
18–34	5473 921 (78.2)	6166 (73.2)	123 (69.9)	6043 (73.3)
>34	1364 963 (19.5)	2187 (26.0)	51 (29.0)	2136 (25.9)
Race/ethnicity				
Hispanic	3492 824 (49.9)	3600 (42.8)	72 (40.9)	3528 (42.8)
Non-Hispanic				
Asian	942 201 (13.5)	1095 (13.0)	18 (10.2)	1077 (13.1)
Black	346 220 (4.9)	782 (9.3)	31 (17.6)	751 (9.1)
Other	335 228 (4.8)	611 (7.3)	12 (6.8)	599 (7.3)
White	1887 861 (27.0)	2334 (27.7)	43 (24.4)	2291 (27.8)
Expected payer for delivery				
Private	3337 678 (47.7)	4756 (56.5)	74 (42.1)	4682 (56.8)
Public	3249 908 (46.4)	3217 (38.2)	93 (52.8)	3124 (37.9)
Self-pay	182 340 (2.6)	59 (0.7)	*	58 (0.7)
Other	234 408 (3.4)	390 (4.6)	*	382 (4.6)
Maternal education (years)				
<12	1456 664 (20.8)	901 (10.7)	31 (17.6)	870 (10.6)
12	1714 319 (24.5)	1919 (22.8)	43 (24.4)	1876 (22.8)
>12	3549 988 (50.7)	5237 (62.2)	90 (51.1)	5147 (62.4)
Prepregnancy BMI (kg/m^2^)				
<18.5	249 080 (3.6)	344 (4.1)	*	340 (4.1)
18.5 to <25	2715 634 (38.8)	3392 (40.3)	72 (40.9)	3320 (40.3)
25 to <30	1508 158 (21.5)	1841 (21.9)	43 (24.4)	1798 (21.8)
≥30	1296 228 (18.5)	1772 (21.0)	31 (17.6)	1741 (21.1)
Hypertension				
Pre-existing	135 417 (1.9)	705 (8.4)	26 (14.8)	679 (8.2)
Gestational	196 633 (2.8)	367 (4.4)	*	358 (4.3)
Pre-eclampsia	262 565 (3.8)	1088 (12.9)	55 (31.3)	1033 (12.5)
Diabetes				
Pre-existing	74 660 (1.1)	189 (2.2)	12 (6.8)	177 (2.2)
Gestational	624 667 (8.9)	771 (9.2)	15 (8.5)	756 (9.2)
Hyperlipidaemia	25 212 (0.4)	100 (1.2)	*	96 (1.2)
Smoking during pregnancy	147 811 (2.1)	421 (5.0)	17 (9.7)	404 (4.9)
Alcohol during pregnancy	18 639 (0.3)	52 (0.6)	*	49 (0.6)
Drug use during pregnancy	116 567 (1.7)	357 (4.2)	20 (11.4)	337 (4.1)
Depression	132 821 (1.9)	597 (7.1)	21 (11.9)	576 (7.0)

* Not displayed when n<11.

APS, antiphospholipid syndrome; ARD, autoimmune rheumatic disease; BMI, body mass index; CVE, cardiovascular event.

**Table 2:** Maternal and neonatal infant outcomes in pregnant individuals without an ARD or APS, and among those with SLE (overall, with LN, with APS)

	No ARD/APS (reference)	SLE		
		Overall SLE	SLE with LN	SLE with APS
	n (%)	n (%)	n (%)	n (%)
Sample	7004 334 (100.0)	8422 (100.0)	903 (100.0)	513 (100)
**Maternal outcomes**				
Maternal CVEs	17 130 (0.2)	176 (2.1)	52 (5.8)	40 (7.8)
Non-cardiac SMM	42 147 (0.6)	275 (3.3)	99 (11.0)	28 (5.5)
Acute renal failure	3052 (0.0)	100 (1.2)	71 (7.9)	14 (2.7)
Acute respiratory distress syndrome	3755 (0.1)	38 (0.5)	13 (1.4)	*
Disseminated intravascular coagulation	5074 (0.1)	35 (0.4)	*	*
Amniotic fluid embolism	119 (0.0)	*	*	*
Blood transfusion	25 725 (0.4)	147 (1.8)	51 (5.7)	12 (2.3)
Severe anaesthesia complications	312 (0.0)	*	*	*
Sepsis	8252 (0.1)	61 (0.7)	23 (2.6)	*
Hysterectomy	2599 (0.0)	12 (0.1)	*	*
Temporary tracheostomy	125 (0.0)	*	*	*
Ventilation	895 (0.0)	*	*	*
Maternal ICU at delivery	6077 (0.1)	39 (0.5)	15 (1.7)	*
Maternal re-admission	331 229 (4.7)	1205 (14.3)	225 (24.9)	121 (23.6)
Maternal death in first year after birth	1765 (0.03)	25 (0.3)	*	*
**Infant outcomes**				
PTB	499 733 (7.1)	1800 (21.4)	312 (45.6)	163 (31.8)
Early (<32 WGA)	50 859 (0.7)	340 (4.0)	129 (14.3)	42 (8.2)
Moderate (32 to <34 WGA)	49 557 (0.7)	219 (2.6)	61 (6.8)	22 (4.3)
Late (34 to <37 WGA)	390 234 (5.6)	1191 (14.1)	204 (22.6)	87 (17.0)
SGA	624 470 (8.9)	1471 (17.5)	235 (26.0)	116 (22.6)
Neonatal ICU at birth	367 689 (5.3)	1226 (14.6)	287 (31.8)	119 (23.2)
Infant re-admission	721 572 (10.3)	1004 (11.9)	128 (14.2)	67 (13.1)
Infant death in first year after birth	30 572 (0.4)	79 (0.9)	19 (2.1)	*

* Not displayed when n<11.

APS, antiphospholipid syndrome; ARD, autoimmune rheumatic disease; CVE, cardiovascular event; ICU, intensive care unit; LN, lupus nephritis; PTB, preterm birth; SGA, small for gestational age; SMM, severe maternal morbidity; WGA, weeks of gestational age.

**Table 3:** Counts, unadjusted and adjusted risk ratios of maternal and infant complications among pregnant individuals with SLE with or without LN or APS, based on the presence or absence of a CVE during pregnancy, compared with a reference group of pregnant individuals without an ARD or APS and without CVEs

	N(row %)	N(row %)	Unadjusted relative risk (RR)	Adjusted relative risk (aRR)
Maternal outcomes	No	Yes		
Non-cardiac severe maternal morbidity (SMM)*				
No ARD/APS, without CVE (n=6987 204)	6939 000 (99.3)	41 133 (0.6)	Reference	Reference
No ARD/APS, with CVE (n=17 130)	14 636 (85.4)	1014 (5.9)	11.0 (10.3, 11.7)	8.8 (8.2, 9.4)
SLE without LN or APS (n=7102)	6897 (97.1)	161 (2.3)	3.9 (3.3, 4.5)	3.4 (2.9, 4.0)
SLE without LN or APS, no CVE (n=7004)	6819 (97.4)	152 (2.2)	3.7 (3.2, 4.3)	3.2 (2.8, 3.8)
SLE without LN or APS, CVE (n=98)	78 (79.6)	†	‡	‡
SLE with LN (n=903)	796 (88.2)	99 (11.0)	18.8 (15.4, 22.9)	12.7 (10.4, 15.4)
SLE with LN, no CVE (n=851)	761 (89.4)	84 (9.9)	16.9 (13.6, 20.9)	11.4 (9.2, 14.2)
SLE with LN, CVE (n=52)	35 (67.3)	15 (28.9)	50.9 (30.7, 84.5)	31.5 (19.0, 52.2)
SLE with APS (n=513)	473 (92.2)	28 (5.5)	9.5 (6.5, 13.7)	5.1 (3.5, 7.4)
SLE with APS, no CVE (n=473)	443 (93.7)	20 (4.2)	7.3 (4.7, 11.4)	3.9 (2.5, 6.0)
SLE with APS, CVE (n=40)	30 (20.0)	†	‡	‡
Maternal ICU utilisation at delivery				
No ARD/APS, without CVE (n=6987 204)	6981 884 (99.9)	5320 (0.1)	Reference	Reference
No ARD/APS, with CVE (n=17 130)	16 373 (95.6)	757 (4.4)	58.0 (53.8, 62.6)	47.5 (43.8, 51.4)
SLE without LN or APS (n=7102)	7083 (99.7)	19 (0.3)	3.5 (2.2, 5.5)	2.7 (1.7, 4.2)
SLE without LN or APS, no CVE (n=7004)	6990 (99.8)	14 (0.2)	2.6 (1.6, 4.5)	2.0 (1.2, 3.4)
SLE without LN or APS, CVE (n=98)	93 (94.9)	†	‡	‡
SLE with LN (n=903)	888 (98.3)	15 (1.6)	21.8 (13.1, 36.2)	12.2 (7.4, 20.3)
SLE with LN, no CVE (n=851)	840 (98.7)	11 (1.3)	17.0 (9.4, 30.7)	9.6 (5.3, 17.3)
SLE with LN, CVE (n=52)	48 (92.3)	†	‡	‡
SLE with APS (n=513)	506 (98.5)	†	‡	‡
SLE with APS, no CVE (n=473)	467 (98.7)	†	‡	‡
SLE with APS, CVE (n=40)	39 (97.5)	†	‡	‡
Maternal re-admission				
No ARD/APS, without CVE (n=6987 204)	6658 338 (95.3)	328 866 (4.7)	Reference	Reference
No ARD/APS, with CVE (n=17 130)	14 767 (86.2)	2363 (13.8)	2.9 (2.8, 3.1)	2.4 (2.3, 2.5)
SLE without LN or APS (n=7102)	6212 (87.5)	890 (12.5)	2.7 (2.5, 2.8)	2.4 (2.2, 2.6)
SLE without LN or APS, no CVE (n=7004)	6135 (87.6)	869 (12.4)	2.6 (2.5, 2.8)	2.4 (2.2, 2.5)
SLE without LN or APS, CVE (n=98)	77 (78.6)	21 (21.4)	4.6 (3.0, 7.0)	3.2 (2.1, 4.8)
SLE with LN (n=903)	678 (75.1)	225 (24.9)	5.3 (4.6, 6.0)	3.8 (3.3, 4.3)
SLE with LN, no CVE (n=851)	646 (76.9)	205 (24.1)	5.1 (4.5, 5.9)	3.6 (3.2, 4.2)
SLE with LN, CVE (n=52)	32 (61.5)	20 (38.5)	8.2 (5.3, 12.7)	5.4 (3.5, 8.4)
SLE with APS (n=513)	392 (76.4)	121 (23.6)	5.0 (4.2, 6.0)	3.0 (2.5, 3.6)
SLE with APS, no CVE (n=473)	368 (77.8)	105 (22.2)	4.7 (3.9, 5.7)	2.8 (2.3, 3.4)
SLE with APS, CVE (n=40)	24 (60.0)	16 (40.0)	8.5 (5.2, 13.9)	6.0 (3.7, 9.8)
Maternal death during or in the first year of delivery				
No ARD/APS, without CVE (n=6987 204)	6985 714 (100.0)	1490 (0.0)	Reference	Reference
No ARD/APS, with CVE (n=17 130)	16 855 (98.4)	275 (1.6)	75.3 (66.2, 85.6)	64.9 (56.8, 74.1)
SLE without LN or APS (n=7102)	7086 (99.8)	16 (0.2)	10.6 (6.5, 17.3)	7.9 (4.8, 12.8)
SLE without LN or APS, no CVE (n=7004)	6990 (99.8)	14 (0.2)	9.4 (5.5, 15.9)	7.0 (4.1, 11.9)
SLE without LN or APS, CVE (n=98)	96 (98.0)	†	‡	‡
SLE with LN (n=903)	894 (99.0)	†	‡	‡
SLE with LN, no CVE (n=851)	846 (99.4)	†	‡	‡
SLE with LN, CVE (n=52)	48 (92.3)	†	‡	‡
SLE with APS (n=513)	512 (99.8)	†	‡	‡
SLE with APS, no CVE (n=473)	473 (100.0)	†	‡	‡
SLE with APS, CVE (n=40)	39 (97.5)	†	‡	‡
Infant outcomes			‡	‡
Preterm birth				
No ARD/APS, without CVE (n=6987 204)	6490 789 (92.9)	496 415 (7.1)	Reference	Reference
No ARD/APS, with CVE (n=17 130)	13 812 (80.6)	3318 (19.4)	2.7 (2.6, 2.8)	2.2 (2.1, 2.3)
SLE without LN or APS (n=7102)	5819 (81.9)	1283 (18.1)	2.5 (2.4, 2.7)	2.3 (2.2, 2.4)
SLE without LN or APS, no CVE (n=7004)	5755 (82.2)	1249 (17.8)	2.5 (2.4, 2.7)	2.3 (2.1, 2.4)
SLE without LN or APS, CVE (n=98)	64 (65.3)	34 (34.7)	4.9 (3.5, 6.8)	3.4 (2.4, 4.7)
SLE with LN (n=903)	491 (54.4)	412 (45.6)	6.4 (5.8, 7.1)	4.6 (4.1, 5.0)
SLE with LN, no CVE (n=851)	477 (56.1)	374 (44.0)	6.2 (5.6, 6.8)	4.4 (4.0, 4.9)
SLE with LN, CVE (n=52)	14 (26.9)	38 (73.1)	10.3 (7.5, 14.1)	6.8 (4.9, 9.3)
SLE with APS (n=513)	350 (68.2)	163 (31.8)	4.5 (3.8, 5.2)	2.7 (2.3, 3.2)
SLE with APS, no CVE (n=473)	331 (70.0)	142 (30.0)	4.2 (3.6, 5.0)	2.5 (2.1, 3.0)
SLE with APS, CVE (n=40)	19 (47.5)	21 (52.5)	7.4 (4.8, 11.3)	5.3 (3.4, 8.1)
Small-for-gestational-age infant§				
No ARD/APS, without CVE (n=6987 204)	5924 065 (84.8)	622 522 (8.9)	Reference	Reference
No ARD/APS, with CVE (n=17 130)	12 308 (71.9)	1948 (11.4)	1.4 (1.4, 1.5)	1.3 (1.2, 1.4)
SLE without LN or APS (n=7102)	4940 (69.6)	1144 (16.1)	2.0 (1.9, 2.1)	1.8 (1.7, 1.9)
SLE without LN or APS, no CVE (n=7004)	4889 (68.8)	1126 (16.1)	2.0 (1.9, 2.1)	1.8 (1.7, 1.9)
SLE without LN or APS, CVE (n=98)	51 (52.0)	18 (18.4)	2.7 (1.7, 4.4)	2.3 (1.5, 3.7)
SLE with LN (n=903)	347 (38.4)	235 (26.0)	4.2 (3.7, 4.8)	3.3 (2.9, 3.7)
SLE with LN, no CVE (n=851)	340 (40.0)	218 (25.6)	4.1 (3.6, 4.7)	3.2 (2.8, 3.6)
SLE with LN, CVE (n=52)	†	17 (32.7)	‡	‡
SLE with APS (n=513)	274 (53.4)	116 (22.6)	3.1 (2.6, 3.8)	2.2 (1.9, 2.7)
SLE with APS, no CVE (n=473)	105 (22.2)	105 (22.2)	3.0 (2.5, 3.7)	2.2 (1.8, 2.6)
SLE with APS, CVE (n=40)	14 (35.0)	11 (27.5)	4.6 (2.6, 8.4)	3.4 (1.9, 6.1)
Infant NICU stay				
No ARD/APS, without CVE (n=6987 204)	6622 167 (94.8)	365 037 (5.2)	Reference	Reference
No ARD/APS, with CVE (n=17 130)	14 478 (84.5)	2652 (15.5)	3.0 (2.9, 3.1)	2.4 (2.3, 2.5)
SLE without LN or APS (n=7102)	6237 (87.8)	865 (12.2)	2.3 (2.2, 2.5)	2.1 (2.0, 2.2)
SLE without LN or APS, no CVE (n=7004)	6162 (88.0)	842 (12.0)	2.3 (2.2, 2.5)	2.1 (1.9, 2.2)
SLE without LN or APS, CVE (n=98)	75 (76.5)	23 (23.5)	4.5 (3.0, 6.8)	3.1 (2.0, 4.6)
SLE with LN (n=903)	616 (68.2)	287 (31.8)	6.1 (5.4, 6.8)	4.3 (3.8, 4.8)
SLE with LN, no CVE (n=851)	597 (70.2)	254 (29.9)	5.7 (5.0, 6.5)	4.0 (3.6, 4.6)
SLE with LN, CVE (n=52)	19 (36.5)	33 (63.5)	12.1 (8.6, 17.1)	7.9 (5.6, 11.2)
SLE with APS (n=513)	394 (76.8)	119 (23.2)	4.4 (3.7, 5.3)	2.6 (2.2, 3.1)
SLE with APS, no CVE (n=473)	367 (77.6)	106 (22.4)	4.3 (3.5, 5.2)	2.5 (2.1, 3.0)
SLE with APS, CVE (n=40)	27 (67.5)	13 (32.5)	6.2 (3.6, 10.7)	4.4 (2.5, 7.5)
Infant re-admission				
No ARD/APS, without CVE (n=6987 204**)**	6267 683 (89.7)	719 621 (10.3)	Reference	Reference
No ARD/APS, with CVE (n=17 130)	15 079 (88.0)	2051 (12.0)	1.2 (1.1, 1.2)	1.2 (1.1, 1.2)
SLE without LN or APS (n=7102)	6281 (88.4)	821 (11.6)	1.1 (1.0, 1.2)¶	1.1 (1.1, 1.2)
SLE without LN or APS, no CVE (n=7004)	6201 (88.5)	803 (11.5)	1.1 (1.0, 1.2)¶	1.1 (1.0, 1.2)¶
SLE without LN or APS, CVE (n=98)	80 (81.6)	18 (18.4)	1.8 (1.1, 2.8)	1.8 (1.1, 2.9)
SLE with LN (n=903)	775 (85.8)	128 (14.2)	1.4 (1.2, 1.6)	1.4 (1.2, 1.7)
SLE with LN, no CVE (n=851)	732 (86.0)	119 (14.0)	1.4 (1.1, 1.6)	1.4 (1.1, 1.6)
SLE with LN, CVE (n=52)	43 (82.7)	†	‡	‡
SLE with APS (n=513)	446 (86.9)	67 (13.1)	1.3 (1.0, 1.6)	1.3 (1.0, 1.6)¶
SLE with APS, no CVE (n=473)	409 (86.5)	64 (13.5)	1.3 (1.0, 1.7)¶	1.3 (1.0, 1.7)¶
SLE with APS, CVE (n=40)	37 (92.5)	†	‡	‡
Infant death in the first year				
No ARD/APS, without CVE (n=6987 204)	6956 822 (99.6)	30 382 (0.4)	Reference	Reference
No ARD/APS, with CVE (n=17 130)	16 940 (98.9)	190 (1.1)	2.6 (2.2, 2.9)	2.2 (1.9, 2.6)
SLE without LN or APS (n=7102)	7048 (99.2)	54 (0.8)	1.7 (1.3, 2.3)	1.6 (1.2, 2.1)
SLE without LN or APS, no CVE (n=7004)	6952 (99.3)	52 (0.7)	1.7 (1.3, 2.2)	1.6 (1.2, 2.1)
SLE without LN or APS, CVE (n=98)	96 (98.0)	†	‡	‡
SLE with LN (n=903)	884 (97.9)	19 (2.1)	4.8 (3.1, 7.6)	3.7 (2.4, 5.9)
SLE with LN, no CVE (n=851)	834 (98.0)	17 (2.0)	4.6 (2.9, 7.4)	3.6 (2.2, 5.7)
SLE with LN, CVE (n=52)	50 (96.2)	†	‡	‡
SLE with APS (n=513)	504 (98.3)	†	‡	‡
SLE with APS, no CVE (n=473)	465 (98.3)	†	‡	‡
SLE with APS, CVE (n=40)	39 (97.5)	†	‡	‡

aRR adjusted using a Propensity Score for lupus created from age, race/ethnicity, primary payer, maternal education, prepregnancy body mass index, clinical comorbidities (pre-existing and pregnancy-induced diabetes mellitus and hypertension, hyperlipidaemia, depression, alcohol, tobacco and drug use during pregnancy)

* Versus no SMM (cardiac or non-cardiac).

† Not displayed when n<11.

‡ Not calculated when n<11.

§ Versus appropriate for gestational age.

¶ p<0.05.

APS, antiphospholipid syndrome; ARD, autoimmune rheumatic disease; CVE, cardiovascular event; ICU, intensive care unit; LN, lupus nephritis; NICU, neonatal intensive care unit; SMM, severe maternal morbidity.

In a sensitivity analysis, pregnancy complications were examined separately for SLE pregnancies with and without CVEs, further stratifying the events by the most common subgroup, that is, VTE-only versus non-VTE CVEs. Formal statistical testing was not conducted in the other subgroups due to the small number of events. All analyses were performed using Statistical Analysis Software V.9.4 (Cary, North Carolina, USA).

### Patients and the public

Patients or the public were not involved in the design, or conduct, or reporting, or dissemination plans of our research.

## Results

The sample included 7004 334 liveborn singletons from individuals without ARDs or APS, and 8422 from SLE. Among 8422 pregnant individuals with SLE, 176 (2.1%) experienced acute CVEs during pregnancy. Pregnant individuals with SLE had a higher proportion of individuals with traditional cardiovascular risk factors such as hypertension, hyperlipidaemia, depression and substance use during pregnancy. When comparing pregnant individuals with SLE who experienced CVEs and those who did not, those with CVEs were more likely to be non-Hispanic black (17.6% vs 9.1%), have public insurance (52.8% vs 37.9%), and less likely to have education beyond 12th grade (51.1% vs 62.4%). They also had a higher prevalence of pre-existing hypertension (14.8% vs 8.2%), pre-eclampsia (31.3% vs 12.5%), tobacco use (9.7% vs 4.9%), drug use (11.4% vs 4.1%) and depression (11.9% vs 7%) (table 1).

Of the 8422 pregnant individuals with SLE, 903 (10.7%) and 513 (6.1%) had LN and APS, respectively. CVEs complicated 17 130 (0.2%) of pregnancies in individuals without ARDs or APS, 176 (2.1%) with SLE, including 52 (5.8%) with LN and 40 (7.8%) with APS (table 2).

Non-cardiac severe maternal morbidity occurred in 275 (3.3%) of pregnant women with SLE, and in 99 (11.0%) of SLE with LN, compared with 42 147 (0.6%) of pregnancies without ARDs or APS. Maternal ICU use during delivery and postdelivery maternal re-admissions were notably high in SLE with LN, at 1.7% and 24.9%, respectively, compared with 0.1% and 4.7% in those without an ARD or APS (table 2). PTB occurred in 1800 (21.4%) of pregnant women with SLE, with higher occurrence for those with APS or LN (163 (31.8%) and 312 (45.6%), respectively), compared with 499 733 (7.1%) in those without an ARD or APS. SGA infants were born to 1471 (17.5%) of pregnant individuals with SLE, with higher occurrence for SLE with APS or LN (116 (22.6%) and 235 (26.0%), respectively), compared with 624 470 (8.9%) of pregnancies in the reference group. Likewise, NICU use, infant re-admission and infant death in the first year were higher in SLE, particularly those with LN at 31.8%, 14.2% and 2.1%, respectively (table 2).

In the general population of pregnant individuals without ARDs or APS, the presence of CVEs during pregnancy significantly increased the risk of all studied maternal complications with aRRs up to 48-fold to 65-fold for maternal ICU admission and mortality in the first year after delivery. Similarly, CVEs increased the adverse infant outcomes by 1.2-fold to 2.4-fold (table 3).

Compared with pregnant individuals without an ARD or APS and without CVEs (reference group), the risks of maternal complications were higher in those with SLE, regardless of APS, LN or CVEs during pregnancy. In SLE, the risk of non-cardiac severe maternal morbidity was higher by 3.2-fold to 31.5-fold, maternal ICU admission by 2.0-fold to 12.2-fold, and maternal re-admission by 2.4-fold to 6.0-fold. The risk of maternal death in the first year was 7.9-fold for SLE without LN or APS but could not be assessed for SLE subgroups with LN, APS or CVEs due to small sample sizes. Whenever the sample size permitted risk estimation for SLE subgroups, the highest risks for adverse maternal outcomes were observed for SLE with LN or APS, particularly when complicated by CVEs (table 3).

Similar results were noted for infant outcomes. Compared with the reference group without an ARD or APS and without CVEs, SLE increased the risk of PTB by 2.3-fold to 6.8-fold, and SGA by 1.8-fold to 3.4-fold, with highest risks seen in SLE with LN or APS, particularly when pregnancy was complicated by CVEs. The risk of NICU admission was 4.0-fold higher in SLE with LN without CVEs and 7.9-fold when CVEs occurred during pregnancy. The risk of infant death was 1.6-fold higher for SLE without LN or APS and 3.6-fold for SLE with LN, even when not complicated by CVEs (table 3).

In the sensitivity analysis, adverse outcomes were more frequent in SLE pregnancies with CVEs than in those without. Non-cardiac severe maternal morbidity and maternal re-admission occurred in 15.3% and 28.4% of SLE pregnancies with CVEs, compared with 3.0% and 14.0% in those without. Similarly, PTB (47.2% vs 20.8%), SGA infants (22.7% vs 17.4%) and infant NICU stays (34.1% vs 14.1%) were notably higher when SLE pregnancies were complicated by CVEs. Non-VTE events in SLE were associated with higher rates of adverse outcomes: maternal re-admission (34.0% vs 20.0%), PTB (51.9% vs 40.0%) and infant NICU stays (38.7% vs 27.1%) compared with VTE-only events (table 4).

**Table 4:** Counts of maternal and infant complications among pregnant individuals with SLE with or without CVEs during pregnancy, stratified by category of CVEs

	SLE without CVE	SLE with CVE		
		Overall	VTE only CVE	Non-VTE only CVE
	N (%)	N (%)	N (%)	N (%)
Sample	8246	176	70	106
Maternal outcomes				
Non-cardiac severe maternal morbidity	248 (3.0)	27 (15.3)	*	20 (18.9)
Maternal ICU utilisation at delivery	30 (0.4)	*	*	*
Maternal re-admission	1155 (14.0)	50 (28.4)	14 (20.0)	36 (34.0)
Maternal death during or in the first year of delivery	19 (0.2)	*	*	*
Infant outcomes				
PTB	1717 (20.8)	83 (47.2)	28 (40.0)	55 (51.9)
SGA	1431 (17.4)	40 (22.7)	17 (24.3)	23 (21.7)
Infant NICU stay	1166 (14.1)	60 (34.1)	19 (27.1)	41 (38.7)
Infant re-admission	974 (11.8)	30 (17.1)	13 (18.6)	17 (16.0)
Infant death in the first year	75 (0.9)	*	*	*

* n<11.

CVE, cardiovascular event; ICU, intensive care unit; NICU, neonatal intensive care unit; PTB, preterm birth; SGA, small for gestational age; VTE, venous thromboembolism.

## Discussion

In all SLE subgroups, including those lacking LN or APS, we found significantly increased risks of selected adverse pregnancy outcomes compared with the reference group without an ARD or APS and without CVEs. APS or LN in SLE contributed to incremental risks for adverse outcomes above SLE without those manifestations. Although the absolute numbers of CVEs were low, the combination of LN or APS with CVEs yielded the highest point estimates for multiple maternal and infant adverse outcomes including severe maternal morbidity, maternal postdelivery re-admission, PTB, SGA and NICU use. The presence of CVEs was also significantly associated with increased risks of all studied adverse maternal and infant outcomes in pregnant individuals without an ARD or APS. In instances where we were able to calculate the risks of adverse outcomes in SLE subgroups with CVEs, the risks exceeded those of non-ARD/APS pregnancies with similar CVEs.

Our findings of increased risk of adverse maternal and infant outcomes in SLE align with existing literature.^1^,^3^ Additionally, SLE is independently associated with an increased risk of maternal CVEs, particularly when LN or APS are present.^10^ While prior studies have established that adverse pregnancy outcomes increase future cardiovascular disease risk and disease-related mortality for the mother,^11^,^13^ our study provides insights into how maternal CVEs influence pregnancy complications, particularly in SLE.

Adverse pregnancy outcomes can significantly impact maternal and infant health, well-being and mortality. Pregnancy complications such as pre-eclampsia and short-term infant outcomes such as PTB, SGA and NICU use have been more frequently studied in relation to SLE. However, less focus has been placed on maternal outcomes such as severe maternal morbidity and ICU use, and longer-term outcomes, including 1 year re-admissions and mortality. For instance, we observed that the risk of non-cardiac severe maternal morbidity in SLE, compared with non-ARD/APS pregnancies without CVEs, increased from 3.2-fold in SLE without LN or APS to 3.9-fold in SLE with APS, and 11.4-fold in SLE with LN, when not associated with CVEs. This risk sharply increased to 31.5-fold in SLE with LN when pregnancy was complicated by CVEs. Our results underscore the importance of disease severity but also highlight the significant impact of CVEs on maternal outcomes. Similar results were observed for infant outcomes, where the highest risk estimates for PTB (6.8-fold) and infant NICU stay (7.9-fold) were for SLE with LN pregnancies complicated by CVEs. The risk estimates for infant re-admissions and deaths within the first year were also higher in SLE with APS or LN. However, the low number of observations in these categories precluded assessment of the impact of CVEs on these outcomes.

This retrospective cohort study using an administrative database was subject to inherent limitations which included reliance on ICD codes used for billing, potential misclassification of diagnoses and under-recording of covariates, including cardiovascular risk factors, based on hospitalisation data. In previous work, sensitivity and specificity estimates for ICD-9 codes in administrative data exceeded 90% for most ARDs, with a lower specificity of 72.5% for SLE.^18^ When restricted to hospitalisation data, notably lower sensitivities have been observed, with sensitivity for SLE ranging from 41% to 68%, for example.^19 20^ In a Norwegian study, the use of ≥1 ICD-10 code to identify incident SLE was associated with a low positive predictive value of 0.47, partly attributed to reclassification of cases initially coded as SLE to other rheumatic conditions.^21^ However, none of these previous studies assessed sensitivity and specificity in pregnant women. The SOMI database used for this study includes hospitalisation codes only covering the year before to a year after delivery, primarily from birth admissions, and validation studies for this scenario are lacking. While requiring >1 ICD code could improve the predictive accuracy, it may lead to under-reporting of SLE by excluding individuals who did not seek care in a hospital-based setting in the year surrounding delivery. Nevertheless, the prevalence of pregnant women with SLE in this data set (0.12% of overall deliveries) aligns with rates observed among women of reproductive age and those with delivery-related admissions.^322^,^24^ The use of more than two ICD-9 codes for SLE combined with renal ICD codes has demonstrated a positive predictive value of 80% for identifying LN in administrative data. However, the accuracy of using ≥1 ICD code, and specifically in pregnancy, is not known.^25^ The absence of specific codes for secondary APS in SLE, along with non-utilisation of non-specific codes (eg, coagulation disorders and elevated antibody titres), may have led to an underestimation of SLE-associated APS in our study. On the other hand, administrative codes from inpatient settings generally have moderate to high sensitivity and specificity estimates (>80%) for acute/incident CVEs.^26^,^29^

Although risks in our study were adjusted for traditional risk factors, there was a lack of information on SLE disease activity including chronicity and activity of LN, CVEs in previous pregnancies, and medications, all of which could influence both cardiovascular diseases and adverse pregnancy outcomes. Pregnant women with SLE who experience CVEs likely represent a subgroup with a greater comorbidity burden, more active SLE and high-dose glucocorticoid use, all of which may contribute to increased risks of adverse outcomes.^30 31^ Furthermore, our study broadly defined CVEs to include various thromboembolic and cardiovascular conditions associated with SLE, but the low absolute number of events for any specific condition limited our ability to perform statistical comparisons within each subgroup. However, in a sensitivity analysis, CVEs, particularly non-VTE events in SLE pregnancies, were associated with numerically higher rates of adverse maternal and infant outcomes. Records in the SOMI database are structured by infant rather than mother; thus, each pregnancy was analysed independently without linkage among siblings. As a result, the lack of independence for repeat pregnancies from the same mother could not be addressed. With respect to previous pregnancy history, while some data in SOMI are available on prior pregnancies, no information was available for specific diagnoses, for example, CVEs in a prior pregnancy. Further, due to not accounting for multiple comparisons, there is a potential for type 1 error in the interpretations. Strengths include the large size and complete population-based sample which was achieved through the linkage of vital statistics records in the state of California to maternal discharge records for all births in California spanning a 15-year period. Furthermore, we were able to examine the influence of CVEs during pregnancy on a broad range of maternal and infant outcomes across different SLE subpopulations.

Our study findings help to individualise the risks of pregnancy complications for various SLE subpopulations and emphasise the need for targeted strategies to mitigate the burden of adverse pregnancy outcomes in SLE. Future prospective studies should precisely assess the timing of the CVEs relative to the stage of pregnancy and the outcomes of interest and evaluate the impact of SLE medications on cardiovascular and obstetric risks. Prospective studies in diverse populations are needed to validate our findings and identify population-specific risk factors to inform screening and interventions that reduce acute CVEs and poor pregnancy outcomes. Additionally, the broader implications of maternal CVEs on later disease risk in childhood and adulthood need further exploration. Despite the low absolute numbers, given the highest risks of severe maternal and infant morbidity in association with CVEs, heightened surveillance and preventive measures during prenatal care are important in this high-risk group.

## Supplementary material

10.1136/lupus-2025-001507 online supplemental table 1

## Data Availability

All data relevant to the study are included in the article or uploaded as supplementary information.
