# Supplementary material for: Adverse pregnancy outcomes across SLE subgroups: significance of cardiovascular events
Source: Lupus Sci Med. 2025 Apr 29;12(1):e001507. doi: 10.1136/lupus-2025-001507 (PMC12049864; doi:10.1136/lupus-2025-001507)
Supplement: online supplemental table 1 [file lupus-12-1-s001.docx]

| **Supplementary Table 1**. ICD 9 and 10 codes used to identify autoimmune rheumatic diseases (ARDs) and antiphospholipid syndrome (APS) | | |
| --- | --- | --- |
|  | ICD-9-CM codes | ICD-10-CM codes |
| **APS** | 289.81 | D68.312, D68.61, D68.62 |
| **Systemic lupus erythematosus (SLE)** | 710.0 | M32.1x, M32.8, M32.9 |
| **Lupus nephritis** | SLE **plus** (580.00-586.00 and 791.0) | M32.14, M32.15,  SLE **plus** (N17-N19, N00-N06, N08, R80.9) |
| **SLE with APS** | SLE **plus** (289.81) | SLE **plus** (D68.312, D68.61, D68.62) |
| **Rheumatoid arthritis** | 714.0, 714.1, 714.2 | M05.x (MO5–MO5.9), MO6.0x, MO6.8x, and MO6.9 |
| **Spondyloarthritis** | | |
| PsA | 696.0 | L40.5x (L40.50-L40.59) |
| AS | 720.0 | M45.x |
| **Sjogren’s syndrome** | 710.2 | M35.**0**x |
| **Other rheumatic conditions** | | |
| **Systemic sclerosis** | 710.1 | M34.x |
| **Inflammatory myositis** |  |  |
| Dermatomyositis | 710.3 | M33.0x, M33.1x, M33.9x |
| Polymyositis | 710.4 | M33.2x |
| **Vasculitides** |  |  |
| Polyarteritis nodosa | 446.0 | M30.0 |
| Granulomatosis with polyangiitis, Eosinophilic granulomatosis with polyangiitis, Microscopic polyangiitis | 446.4 | M30.1, M31.3 (M31.30, M31.31), M31.7 |
| Giant cell arteritis | 446.5 | M31.5, M31.6 |
| Takayasu disease | 446.7 | M31.4 |

| **Supplementary Table 2**. ICD codes used to identify cardiovascular events (CVEs) and cardiovascular risk factors. | | |
| --- | --- | --- |
|  | **ICD-9 codes** | **ICD-10 codes** |
| **Cardiovascular events (CVEs)** | | |
| **Acute myocardial infarction (AMI)** | 410 | I21, I22 |
| **Acute cerebrovascular accidents** | | |
| Hemorrhagic stroke | 430, 431 | I60, I61 |
| Ischemic stroke | 433.x1 (433.01, 433.11, 433.21, 33.31, 433.81, 433.91), 434.x1 (434.01, 434.11, 434.91) | I63 |
| **Heart failure (acute or unspecified, not rheumatic)** | 402.01, 402.11, 402.91, 404.01, 404.03, 404.11, 404.13, 404.91, 404.93, 428.0, 428.1, 428.20, 428.21, 428.23, 428.30, 428.31, 428.33, 428.40, 428.41, 428.43, 428.9 | I11.0, I13.0, I13.2, I50.1, I50.20, I50.21, I50.23  I50.30, I50.31, I50.33, I50.40, I50.41, I50.43  I50.8x (except I50.812), I50.9 |
| **Peripartum cardiomyopathy (PPCM)** | 674.5x (674.50 - 674.54) | O90.3 |
| **Inflammatory heart diseases** | | |
| Acute pericarditis | 420 | I30.0, I30.8, I30.9, I32 |
| Acute myocarditis (not septic or toxic) | 422.0, 422.90, 422.91, 422.99, 429.0 | I40.1, I40.8, I40.9, I41, I51.4 |
| **Cardiac dysrhythmias** | | |
| Atrial fibrillation/flutter | 427.3x (427.31, 427.32) | I48 (I48.0, I48.11, I48.19, I48.20, I48.21, I48.3, I48.4, I48.91, I48.92) |
| Ventricular arrhythmias, cardiac arrest | 427.1, 427.4x (472.41, 427.42), 427.5, 798, 798.1, 798.2, 798.9, 799.9 | I46.x (I46.2, I46.8, I46.9), I47.2x (I47.20, I47.21, I47.29), I49.0x (I49.01, I 49.02), R99 |
| Cardiac dysrhythmia, unspecified | 427.9 | I49.9 |
| **Venous thromboembolism (VTE)** | | |
| Pulmonary embolism (not septic) | 415.11, 415.13, 415.19 | I26.02, I26.09, I26.92, I26.93, I26.94, I26.99 |
| Deep vein thrombosis (DVTs) of upper/lower extremities and other sites | Upper extremity DVT: 451.83, 451.84, 451.89, 453.82, 453.83, 453.84, 453.85, 453.87  Lower extremity DVT: 451.1x (451.11, 451.19), 451.2, 451.81, 453.4x (453.40, 453.41, 453.42),  Other sites DVT: 451.9, 453.1, 453.2, 453.3, 453.89, 453.9 | Upper extremity DVT: I82.6 (I82.60x, I82.61x, I82.62 x)  Lower extremity DVT: I80.1 (I80.10-180.13), I80.2 (I80.20x, I80.21x, I80.22x, I80.23x, I80.24x, I80.25x, I80.29x), I80.3, I82.4 (I82.40x, I82.41x, I82.42 x, I82.43 x, I82.44 x, I82.45 x, I82.46 x, I82.49 x, I82.4Yx, I82.4Zx)  Other sites DVT: I80.8, I80.9, I82.1, I82.210, I82.220, I82.290, I82.3, I82.A1x, I82.B1x, I82.890, I82.90 |
| Deep phlebothrombosis and thromboembolism in pregnancy and puerperium | 671.3x, 671.4x, 671.5x, 673.2x | O22.3x, O22.5x, O87.1, O88.2x, O871, O873 |
| **Cardiovascular Risk Factors** | | |
| Diabetes mellitus (DM) | 648.0, 250 | O24.0, O24.1, O24.2, O24.3, E10, E11, E12, E13, E14 |
| Gestational DM | 648.8 | O24.4 |
| Preexisting hypertension complicating pregnancy, childbirth, and puerperium (without superimposed preeclampsia); Unspecified maternal hypertension | 401, 402, 403, 404, 405,  642.0, 642.1, 642.2,  642.9x | I10, I11, I12, I13, I15, I16  O10  O16x  Infant code P00.0 |
| Gestational hypertension without significant proteinuria | 642.3 | O13 |
| Preeclampsia, eclampsia | 642.4, 642.5, 642.6, 642.7 | O11, O14.0, O14.1, O14.9, O15 |
| Hyperlipidemia | 272.0, 272.1, 272.2, 272.3, 272.4 | E78.0, E78.1, E78.2, E78.3, E78.4, E78.5 |
| Smoking during pregnancy | 649.0, 305.1 | Z72.0, F17.2 |
| Alcohol use during pregnancy | 303, 305.0 | F10 |
| Drug use during pregnancy | 648.3, 304, 305.2, 305.3, 305.4, 305.5, 305.6, 305.7, 305.8, 305.9 | F11, F12, F13, F14, F15, F16, F18, F19 |
| Depression | 311, 296.2, 296.3 | F32, F33, F34, F38, F39 |

| **Supplementary Table 3**. ICD-9 and ICD-10 diagnosis and procedure codes and case definitions used to identify non-cardiac Severe Maternal Morbidity (SMM) Indicators | | |
| --- | --- | --- |
|  | **ICD-9 codes** | **ICD-10 codes** |
| Acute renal failure | 584.5, 584.6, 584.7, 584.8, 584.9, 669.3x | N17.x, O90.4 |
| Acute respiratory distress syndrome | 518.5x, 518.81, 518.82, 518.84, 799.1 | J80, J95.1, J95.2, J95.3, J95.82x, J96.0x, J96.2x, J96.9x, R06.03, R09.2 |
| Disseminated intravascular coagulation | 286.6, 286.9, 641.3x, 666.3x | D65, D68.8, D68.9, O45.002, O45.003, O45.009, O45.012, O45.013, O45.019, O45.022, O45.023, O45.029, O45.092, O45.093, O45.099, O46.002, O46.003, O46.009, O46.012, O46.013, O46.019, O46.022, O46.023, O46.029, O46.092, O46.093, O46.099, O67.0, O72.3 |
| Amniotic fluid embolism | 673.1x | O88.112, O88.113, O88.119, O88.12, O88.13 |
| Blood transfusion | 99.0x | 30230H0, 30230K0, 30230L0, 30230M0, 30230N0, 30230P0, 30230R0, 30230T0, 30230H1, 30230K1, 30230L1, 30230M1, 30230N1, 30230P1, 30230R1, 30230T1, 30233H0, 30233K0, 30233L0, 30233M0, 30233N0, 30233P0, 30233R0, 30233T0, 30233H1, 30233K1, 30233L1, 30233M1, 30233N1, 30233P1, 30233R1, 30233T1, 30240H0, 30240K0, 30240L0, 30240M0, 30240N0, 30240P0, 30240R0, 30240T0, 30240H1, 30240K1, 30240L1, 30240M1, 30240N1, 30240P1, 30240R1, 30240T1, 30243H0, 30243K0, 30243L0, 30243M0, 30243N0, 30243P0, 30243R0, 30243T0, 30243H1, 30243K1, 30243L1, 30243M1, 30243N1, 30243P1, 30243R1, 30243T |
| Severe anesthetic complications | 668.0x, 668.1x, 668.2x, 995.4, 995.86 | O29.112–O29.119, O29.122–O29.129, O29.192–O29.199, O29.212–O29.219, O29.292–O29.299, O74.0, O74.1, O74.2, O74.3, O89.0x, O89.1, O89.2, T88.2XXA, T88.3XXA |
| Sepsis | 038.xx, 449, 785.52, 995.91, 995.92, 998.02, 670.2x (after October 1, 2009) | A32.7, A40.x, A41.x, I76, O85, O86.04, R65.20, R65.21, T81.12XA, T81.44XA |
| Hysterectomy | 68.39, 68.49, 68.59, 68.69, 68.79, 68.9 (also include 68.3, 68.4, 68.5, 68.6, 68.7; non-specific codes used frequently) | 0UT90ZL, 0UT90ZZ, 0UT97ZL, 0UT97ZZ |
| Ventilation | 96.70, 96.71, 96.72 | 5A1935Z, 5A1945Z, 5A1955Z |
| Temporary tracheostomy | 31.1 | 0B110F4, 0B113F4, 0B114F4 |
| The ICD-9 and ICD-10 diagnosis and procedure codes for non-cardiac SMM were adapted from the CDC definitions of these SMM indicators  (Source: <https://www.cdc.gov/maternal-infant-health/php/severe-maternal-morbidity/icd.html?CDC_AAref_Val=https://www.cdc.gov/reproductivehealth/maternalinfanthealth/smm/severe-morbidity-ICD.htm>) | | |
